# The Effects of Accumulated Versus Continuous Exercise on Postprandial Glycemia, Insulin, and Triglycerides in Adults with or Without Diabetes: A Systematic Review and Meta-Analysis

**DOI:** 10.1186/s40798-021-00401-y

**Published:** 2022-01-24

**Authors:** Xiaoyuan Zhang, Chen Zheng, Robin S. T. Ho, Masashi Miyashita, Stephen Heung Sang Wong

**Affiliations:** 1 Department of Sports Science and Physical Education, The Chinese University of Hong Kong, Hong Kong, China; 2 Department of Physical Education, Peking University, Bejing, China; 3 Faculty of Sport Sciences, Waseda University, Saitama, Japan

**Keywords:** Accumulated exercise, Continuous exercise, Exercise patterns, Insulin, Postprandial glucose, Triglycerides

## Abstract

**Background:**

Postprandial dysmetabolism, an important cardiovascular disease risk factor, can be improved by exercise. Further systematic review and meta-analysis is needed to compare the effects of accumulated exercise with a single session of energy-matched continuous exercise on postprandial glucose (PPG), insulin, and triglycerides in adults with or without diabetes.

**Methods:**

Eight electronic databases were searched on August 28, 2020, and updated on April 27, 2021. Eligible studies were randomized, quasi-randomized, or non-randomized controlled or crossover trials that evaluated the acute or longitudinal effects of accumulated exercise compared with a single session of energy-matched continuous exercise on PPG, postprandial insulin, and triglycerides in diabetic and non-diabetic adults. Same-day and second-morning effects were assessed separately for acute intervention studies. Subgroup analyses were conducted based on the number of exercise bouts (2–3 bouts or frequent brief bouts (e.g., 1–6 min) throughout the day at 20–60-min intervals (known as physical activity [PA] breaks, ≥ 5 bouts)), exercise intensity, and populations. Risk of bias was assessed using the revised Cochrane risk-of-bias tool for randomized trials. Pooled effects were reported as standardized mean differences (SMD) and 95% CI using a random effects model.

**Results:**

Twenty-seven studies (635 participants) were included. A significant difference was found for same-day PPG control, which favored accumulated exercise over one bout of energy-matched continuous exercise (SMD − 0.36 [95%CI: (− 0.56, − 0.17)], *P* = 0.0002, *I*^2^ = 1%), specifically in accumulated exercise with PA breaks (SMD − 0.36 [95%CI: (− 0.64, − 0.08)], *P* = 0.01, *I*^2^ = 30%), low-moderate intensity exercise (SMD − 0.38 [(95%CI: (− 0.59, − 0.17)], *P* = 0.0005, *I*^2^ = 0%), and in non-diabetic populations (SMD − 0.36 [95%CI: (− 0.62, − 0.10)], *P* = 0.007, *I*^2^ = 16%). No differences were found for same-day postprandial insulin and triglycerides, and second-morning effects (postprandial or fasting glucose, insulin, and triglycerides) between different exercise patterns.

**Conclusion:**

Compared with one session of continuous exercise, accumulated exercise—specifically in subgroups of PA breaks, low-moderate intensity exercises—produced greater acute effects on same-day PPG control for non-diabetic adults. There were no differences between continuous and accumulated patterns of exercise in terms of same-day postprandial insulin and triglycerides, and second-morning effects on all previously mentioned markers. The findings provide additional PA options for PPG control for individuals with limited time or exercise capacity to engage in PA in one session.

Registration: PROSPERO (identification code: CRD42021251325).

**Supplementary Information:**

The online version contains supplementary material available at 10.1186/s40798-021-00401-y.

## Key Points

- In adults with or without diabetes, accumulated exercise produced greater acute effects on same-day postprandial glucose control than one session of energy-matched continuous exercise.
- No differences were found between continuous and accumulated exercise in terms of same-day postprandial insulin and triglycerides, and second-morning effects.
- Accumulated exercise is an effective and feasible alternative for postprandial glucose control for adults with or without diabetes.

## Introduction

Postprandial dysmetabolism, most notably hyperglycemia and hypertriglyceridemia, is an important cardiovascular disease (CVD) risk factor, inducing endothelial dysfunction both independently and cumulatively through oxidative stress [1, 2]. An accumulating body of evidence now suggests that postprandial glucose (PPG) is more closely correlated with microvascular and macrovascular morbidities and cardiovascular mortality than hemoglobin A1c (HbA1c) or fasting glucose [3–5]. The role of exercise in reducing PPG levels [6, 7] and improving glycemic control and overall health [8–10] in diabetic, as well as in healthy, populations has been well established [11, 12]. However, how and when aerobic exercise should be prescribed to optimize glucose control remains controversial [13].

Several studies [14–16] and reviews [17–19] have shown that multiple postprandial bouts of exercise elicit greater improvements to PPG levels compared to a single exercise bout, with some studies indicating that continuous exercise has a comparable [13] or superior [20] effect than accumulated exercise. It should be noted that the two commonly used accumulating exercise patterns in these previous studies were frequent brief bouts (e.g., 1–6 min) throughout the day at 20–60-min intervals (known as physical activity [PA] breaks, ≥ 5 bouts), and three short bouts (e.g., 10–15 min) timed around the main meals at 3–5-h intervals [18].

The World Health Organization PA guidelines [21] recommend that adults accumulate at least 150–300 min of moderate-intensity aerobic PA per week to gain health benefits. As the prevalence of insufficient PA is still very high, identifying the glycemic and lipemic benefits of accumulated exercise may provide an alternative choice for those who do not have sufficient time to exercise in one session. Moreover, accumulating exercise may also induce more interruptions to prolonged sitting.

Previous systematic review and meta-analyses [17, 19] have compared the acute effects of PA breaks and continuous exercise on glucose regulation, finding that when energy expenditure was matched, the former had a greater effect on glucose regulation than the latter. This result may be explained by one-bout exercise likely inducing glucose counterregulation, as evidenced by the elevated glucose levels during exercise compared to uninterrupted sitting [19]. Glucose counterregulation implies the physiological processes of increasing hepatic glucose output via counterregulatory hormones (e.g., glucagon and epinephrine) to prevent or correct hypoglycemia [22]. However, studies with three short bouts (e.g., 10–15 min) of exercise timed around daily main meals were not included in these reviews [17, 19].

Chang et al. [18] included both patterns of accumulated exercise and showed that three short bouts (e.g., 10–15 min) of exercise timed around daily main meals were comparable or superior to a single continuous bout for improving glycemic control in individuals with type 2 diabetes, while the benefits of PA breaks, compared to a single bout of continuous activity, for improving glycemic control were unclear. However, only individuals with diabetes or pre-diabetes were included, only glucose outcomes were examined, and a meta-analysis was not conducted in the above-mentioned review [18]. Moreover, same-day and second-morning effects were not analyzed independently against PPG [17–19]; these effects may produce different outcomes [23]. Thus, it is necessary to further examine the effects of accumulating exercise in multiple short bouts or frequent PA breaks, in comparison to a single continuous bout of exercise, on PPG, postprandial insulin, and triglyceride (TG) responses in diabetic and non-diabetic populations.

Therefore, we conducted a systematic review and meta-analysis to compare the acute and longitudinal effects of accumulating exercise in either multiple short bouts or PA breaks with a single continuous bout of exercise on glucose, insulin, and TG outcomes in healthy adult and diabetic populations.

## Methods

This systematic review follows the Preferred Reporting Items for Systematic Reviews and Meta-Analyses (PRISMA) guidelines [24], and is prospectively registered at the International Prospective Register of Systematic Reviews (PROSPERO) (identification code: CRD42021251325).

### Search Strategy

A systematic search was conducted using the databases MEDLINE, EMBASE, the Cochrane Central Register of Controlled Trials (CENTRAL), Web of Science, SPORTDiscus, Cumulative Index to Nursing and Allied Health Literature (CINAHL), PsycINFO, and ClinicalTrials.gov on August 28, 2020; this search was subsequently updated on April 27, 2021. Search results were imported into Endnote X7 (Thomson Reuters, Toronto, Canada) and duplicates were eliminated using an automated feature. Details of the literature search strategy are available in Additional file 1: Table S1. Furthermore, we manually searched the reference lists of articles included in the final analysis.

### Study Selection

**Fig. 1:**
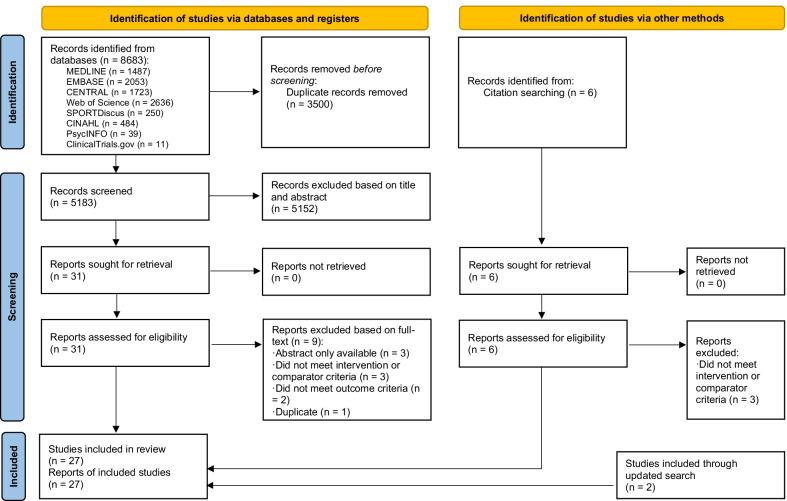
The PRISMA flow diagram

Two reviewers (XZ, CZ) independently screened the articles based on titles and abstracts, followed by a full-text review for eligibility of the inclusion criteria. Any discrepancies were resolved through discussion with a third reviewer (RH). Eligibility of a study was determined according to specific inclusion criteria, as listed below. Briefly, we included any studies that evaluated of multiple bouts of accumulated, compared with a single session of energy-matched continuous, exercise on PPG, postprandial insulin, and TG in diabetic and non-diabetic adults. Non-diabetic adults were generally healthy and without any major health conditions. Both short- and long-term intervention studies were included. A flow diagram of the search and screening process is presented in Fig. 1.

The inclusion criteria of studies are listed below:

1. Study design: Randomized crossover trials, and randomized, quasi-randomized, or non-randomized controlled trials were eligible to be included in this review. Single group, cohort, and cross-sectional trials were excluded.
2. Population: Studies of non-diabetic and diabetic adults (aged 18 + years) were eligible for review. Non-diabetic adults were required to be generally healthy and without any major health conditions (e.g., cancers, heart disease, and chronic obstructive pulmonary disease). Individuals that were overweight/obese or at high risk of disease were also eligible. Diabetic adults included individuals with type 2 diabetes, type 1 diabetes, insulin-dependent diabetes mellitus, and individuals with impaired fasting glucose, impaired glucose tolerance or insulin resistance.
3. Intervention: Studies that conducted accumulated exercise in multiple bouts, with at least 5-min intervals between bouts, throughout a single day were eligible for this review. Both longitudinal exercise training and acute exercise intervention studies were included. Multiple bouts of exercise, either ≥ 10 or ˂10 min per bout, were eligible for inclusion. Intermittent exercises performed in the same exercise session (e.g., high-intensity intermittent exercise (HIIT), characterized by repeated short bouts of high-intensity exercise separated by brief periods of low-intensity activity or rest) with intervals < 5 min were not included as accumulated exercise, but were included as one session of continuous exercise, as listed in “4. Comparison”.
4. Comparison: Studies that compared the above-mentioned accumulated exercise with a single session of energy-matched continuous exercise were included in this review. One session of exercise with intervals < 5 min (e.g., HIIT) was also eligible. Studies comparing accumulated exercise with only a control (e.g., sitting or no exercise) trial or group were excluded. Studies with accumulated exercise that were not energy-matched with continuous exercise were not eligible for this review.
5. Outcomes: Studies that examined one of the following outcomes were eligible for this review: (1) fasting or PPG (area under the curve (AUC), mean glucose or glucose concentration at 2 h, etc.); (2) fasting or postprandial insulin; (3) fasting or postprandial TG; (4) other outcomes related to glucose, insulin, or TG, such as HbA1c, duration in hyperglycemia, and glucose variability.

### Data Extraction

Two reviewers (XZ, CZ) extracted descriptive characteristics and outcomes from each included study in duplicate. Any discrepancies were resolved through discussion with a third reviewer (RH). Study characteristics are summarized in Tables 1 and 2, including first author name, publication year, study design, participant characteristics (number, sex, age, BMI, and inclusion criteria, if any), intervention protocol for accumulated exercise, continuous exercise, and control (if any), and main results. The primary outcome of this review was PPG AUC. The secondary outcomes included 24-h mean glucose, postprandial insulin AUC, postprandial TG AUC, fasting glucose, insulin, and TG. Moreover, glucose variability (e.g., mean amplitude of glycemic excursions), duration in hyperglycemia, and HbA1c were also extracted if available.

**Table 1:** Short-term study characteristics

Studies	Study design	Participants (N(M/F), age [year], BMI [kg/m^2^])	Intervention	Results
*Same-day effect*				
Bailey et al. [44]	Randomized crossover	Sedentary & inactive but healthy adults, *N* = 12 (M5/F7) M: Age: 25.0 ± 5.4; BMI:25.0 ± 3.9; F: Age: 22.6 ± 1.5; BMI: 21.3 ± 3.9	SED-ACT: 10 × 60 s with an interval of 30-min HIIE: 10 × 60 s with 1-min @60Watt active recovery between bouts Cycling @90% V̇O_2_max; @45-min post-breakfast SED: prolonged uninterrupted sitting for 6.5-h	PPG iAUC:↓1.91 mmol/L/6.5-h (SED-ACT vs SED) PPTG iAUC:↓1.02 mmol/L/6.5-h (HIIE vs SED)
Bhammar et al. [52]	Randomized crossover	Overweight/obese, physically inactive adults, *N* = 10 (M5/F5) Age: 32 ± 5; BMI: 30.3 ± 4.6	2-min VIG: 8 × 2-min @79 ± 4% HRmax, every hour, ~ 140 kcal 2-min MOD: 21 × 2-min @53 ± 5% HRmax, every 20-min, ~ 240 kcal 30-min MOD: Start@12:00 h, 1 × 30-min @71 ± 4%HRmax, ~ 230 kcal SIT: 9-h prolonged sitting	Mean 18.7-h glucose: ↓0.4 mmol/L (2-min MOD vs SIT) ↓0.2 mmol/L (2-min VIG vs SIT) 30-min MOD < all other trials
Blankenship et al. [32]	Randomized crossover	T2D, *N* = 30 (M14/F16) Age: 64 ± 8.2 BMI: 31.7 ± 5.4	Break (BR): 12 bouts*1.6, 3.3, 5 min every 30-min for 2-h after each meal, in total 20, 40, 60-min WALK: 1 bout*20, 40, 60-min; after breakfast Intensity @faster than their usual walking speed Control: maintained habitual levels of PA	Daily duration of hyperglycemia: WALK < Control; ↔ BR vs Control Breakfast PPG:↓BR or WALK vs Control Lunch & dinner PPG: ↔ BR and WALK vs Control
DiPietro et al. [61]	Randomized crossover	Inactive older, *N* = 10 Age: 69 ± 6; BMI: 30 ± 5;	Postmeal walking: 15-min walk, @30 min after each meal Sustained walking: 45-min of sustained walking performed @10:30 or 16:30 Intensity @3METs Control day: remain inactive	24-h glycemic:↓in sustained 10:30 A.M. walking & Postmeal walking vs Control day 3-h postdinner glucose: Postmeal walking < All sustained walking
Engeroff et al. [35]	Randomized crossover	Females, *N* = 18 Age: 25.6 ± 2.6 BMI: 21.5 ± 2.0	BREAK: 40-min after breakfast, 5 × 6-min cycling, interval: sitting for 40 min PRE: 30-min cycling pre-breakfast and 4-h sitting Intensity @70% V̇O_2_max CTRL: 4-h sitting	ΔtChol: ↓BREAK vs PRE; ↔ BREAK vs CTRL TG: ↑in BREAK (after vs before trial); ↑in CTRL (after vs before trial); ↔ in PRE (after vs before trial)
Francois et al. [16]	Randomized crossover	Insulin resistance, *N* = 9 (M7/F2) Age: 48 ± 6 BMI: 36 ± 8	Exercise Snacking (ES): 6 × 1-min intense incline walking Composite Exercise Snacking (CES): 6 × 1-min intervals alternating between walking and resistance-based exercise ES & CES: walking @90% HRmax, 30-min before each meal Continuous exercise (CONT): 30-min continuous incline walking @60% HRmax before dinner	Mean 3-h PPG: ↓after breakfast & dinner; ↔ after lunch (ES vs CONT) Mean 24-h, subsequent 24 h PPG:↓ES vs CONT Glycemic control: ↔ CES vs ES
Haxhi et al. [33]	Randomized crossover	T2D, *N* = 9 (M7/F2) Age: 58.2 ± 6.6; BMI: 30.2 ± 3.1	SplitEx: walking 20-min before and after 40-min of a standard lunch ContEx: walking 40-min after 40-min of a standard lunch Intensity @52.2 ± 5.9% HRR CTRL: no exercise	Duration in moderate hyperglycemia after lunch: ↓25.8% SplitEx vs ContEx Duration in hyperglycemia after breakfast: ↓18.1% ContEx vs CTRL 24-h urinary isoprostanes: ↓68% ContEx vs CTRL, ↓63% SplitEx vs CTRL
Holmstrup et al. [45]	Randomized crossover	Obese, *N* = 11 (M8/F3) Mean age: 25 Mean BMI: 34	Intermittent exercise (INT): 12 × 5-min, hourly Morning Exercise (EX): sedentary behavior with 1-h morning exercise Walking @60–65% V̇O_2_peak Sedentary behavior (SED): CTRL	12-h glucose iAUC:↓INT & SED vs EX 12-h insulin iAUC:↓in INT & EX vs SED Insulin production rate:↑in INT vs SED or EX
Kashiwabara et al. [36]	Randomized crossover	Inactive women with hypertriglyceridemia, *N* = 12 Age: 71 ± 5	Multiple: 20 × 90-s, every 15 min with 3 longer breaks over 8-h Continuous: 1 × 30-min Walking @self-selected pace start @40-min after breakfast CTRL: sitting for 8-h	PPTG iAUC: ↓35% in Continuous vs CTRL; ↓33% in Multiple short bouts vs CTRL
Maylor et al. [46]	Randomized crossover	Sedentary and inactive adults, *N* = 14 (7 M/7F) Age: 29 ± 9 BMI: 26.1 ± 5.8	SIT-ACT: 8 × 2-min 32-s, every 1-h, @85% V̇O2R CONT-SIT: 1 × 30-min @60% V̇O_2_R, followed by prolonged sitting (7-h) Exercise on a treadmill, @30-min after breakfast SIT: remained seated	PPTG iAUC:↓ SIT-ACT vs SIT HDL-C iAUC:↑SIT-ACT vs SIT TG, HDL-C iAUC: ↔ CONT-SIT vs SIT or SIT-ACT
Miyashita et al. [37]	Randomized crossover	Inactive postmenopausal women, *N* = 15 Age: 68.8 ± 3.2 BMI: 24.0 ± 2.9	Regular walking: 20 × 1.5-min walking, every 30 min Continuous walking: 1 × 30-min walking Intensity @self-selected pace (3.7 ± 1.1 km/h) @40 min after breakfast; Prolonged sitting: rest in-lab	Serum PPTG iAUC: ↓ 15% Regular walking vs Prolonged sitting ↓ 14% Regular walking vs Continuous walking
Murphy et al. [62]	Randomized crossover	*N* = 10 (M3/F7); Sedentary men: [Age: 46(34–52); BMI: 29.7(25.1–34.5)]; Postmenopausal Sedentary women: [Age: 55(49–66); BMI: 27.2(23.9–33.2)]	Short walk: 3 × 10-min walking before each meal Long walk: 1 × 30-min walking before breakfast Intensity @self-selected pace (5.9 ± 2.6 km/h) CTRL: rest	PPTG:↓Short vs CTRL; ↓Long walks vs CTRL; ↔ Short vs Long walks Postprandial fat oxidation:↑when both walking trials were treated as one condition
Peddie et al. [50]	Randomized crossover	Adults, *N* = 70 (M23/F42) Age: 25.9 ± 5.3 BMI: 23.6 ± 4.0	Regular activity-break: 18 × 1-min 40-s walking, every 30 min over 9-h Physical activity: 1 × 30-min walking, @15-min before 1st meal, then sit 8-h15-min Intensity @60% V̇O_2_max Prolonged sitting: Prolong sitting 9-h	Plasma insulin iAUC:↓Regular activity-break vs Prolonged sitting or Physical activity PPG iAUC:↓Regular activity-break vs Prolonged sitting or Physical activity Plasma PPTG iAUC:↓Physical activity vs Regular activity-break
Shambrook et al. [13]	Randomized crossover	Adults, *N* = 10 (M8/F2) Age: 50.0 ± 12.6 BMI: 29.0 ± 5.4	APPW: 3 × 10-min walking, @30-min after each meal CPPW: 1 × 30-min walking, @30-min after dinner Intensity @55–70% HRR NOEX: no exercise	PPG dinner: ↓ APPW or CPPW vs NOEX ↔ APPW vs CPPW
*Second-day effect*				
Altena et al. [47]	Randomized crossover	Inactive normolipidemic adults, *N* = 18 (7 M/11F) Age: 25.0 ± 7.6; BMI: 23.2 ± 3.4	INT-EX: 3 × 10-min walk, interval: sitting for 20-min (Day 1) CON-EX: 30-min walk (Day 1) All treadmill running @60% V̇O_2_max NOEX: no exercise (Day 1)	PPTG iAUC & iPeak:↓INT-EX vs NOEX; ↔ CON-EX vs INT-EX or NOEX;↑M vs F in INT-EX & CON-EX; ↔ M vs F in NOEX
Baynard et al. [34]	Randomized crossover	T2D women & non-obese women, *N* = 15 T2D: (*n* = 9; Age: 53 ± 6; BMI: 36.1 ± 5.4); CTRL: (*n* = 6; Age: 49 ± 4; BMI: 22.0 ± 1.5)	Multiple bout: 3 × 10-min, @8:00 am, 12:00 pm, and 5:15 pm (Day 1) Single bout: 1 × 30 min @5:00 pm (Day 1) All treadmill running @60–65% V̇O_2_max Control day: no exercise (Day 1)	OGTT glucose response: ↔ 3 conditions within each group; 2-h OGTT glucose (Non-diabetic): ↓ Multiple bout vs Single bout or Control day); 2-h OGTT glucose (T2D): ↑ Multiple bout vs Single bout, ↓ Multiple bout vs Control day
Gill et al. [39]	Randomized crossover	Healthy males, *N* = 18 Age: 30.6 ± 9.0; BMI: 23.1 ± 1.4;	Intermittent: 3 × 30 min running, interval: 3.75-h (Day 1) Continuous: 1 × 90 min running on the afternoon (Day 1) Intensity @60% V̇O_2_max CTRL: no exercise (Day 1)	Fasting plasma TG: ↔ between trials PPTG AUC:↓18.1% Intermittent vs CTRL,↓17.7% Continuous vs CTRL; PPG: ↔ between trials Serum insulin response:↓Intermittent vs CTRL
Kim et al. [40]	Randomized crossover	Healthy recreationally active men, *N* = 9 Age: 24 ± 4	LOW: 9 intermittent walking sessions hourly from 0900 to 1800 h @25% V̇O_2_max (Day 3) MOD: 1 × 60-min running @65% V̇O_2_max (Day 3) from 1700 to 1800 h; CTRL: sitting no exercise control (Day 3)	PPTG iAUC:↓33.6% MOD vs CTRL; ↓19.8% LOW vs CTRL;↓17.2% MOD vs LOW Plasma glucose:↓MOD vs LOW or CTRL Fat oxidation:↑MOD vs LOW or CTRL
Miyashita et al. [43]	Randomized crossover	Healthy recreationally active men, *N* = 10 Age: 25.0 ± 4.1 BMI: 25.4 ± 3.8	ACCU: 10 × 3-min walking, interval: 30-min (Day 1) CONT: 1 × 30-min walking after lunch (Day 1) Intensity @70% V̇O_2_max, finished @Day1 15:30 h CTRL: rest in-lab (Day 1)	Day 2 plasma PPTG iAUC: ↓ACCU vs CTRL;↓CONT vs CTRL
Miyashita [41]	Randomized crossover	Sedentary men, *N* = 8 Age: 26.5 ± 4.2 BMI: 28.9 ± 4.0	ACCU: 10 × 3-min cycling, Interval: 30-min (Day 1) CONT: 1 × 30-min cycling after lunch (Day 1) Intensity @60% HRmax, finished @Day1 15:30 h; CTRL: rest in-lab (Day 1)	Day 2 serum PPTG iAUC: ↓ACCU vs CTRL;↓CONT vs CTRL
Miyashita et al. [42]	Randomized crossover	Men, *N* = 15 Age: 23.4 ± 3.1 BMI: 23.4 ± 2.3	ACCU: 10 × 3-min walking, interval: 30-min (Day 1) CONT: 1 × 30-min walking after lunch (Day 1) Intensity @self-selected pace (~ 42% V̇O_2_max), finished @15:30 h CTRL: rest in-lab (Day 1)	Day 2 plasma PPTG iAUC: ↓ACCU vs CTRL;↓CONT vs CTRL Resting SBP: ↓ACCU vs CTRL;↓CONT vs CTRL
Shambrook et al. [51]	Randomized crossover	Insufficiently active adults, *N* = 10 (M8/F2) Age: 50.0 ± 12.6 BMI: 29.0 ± 5.4	ACC: 3 × 10-min walking, @30-min after each meal (5-d) CONT: 1 × 30-min walking, @30-min after dinner (5-d) Intensity @55–70% HRR NOEX: no exercise (5-d)	OGTT 2-h glucose iAUC: ↔ CONT vs ACC OGTT 2-h insulin iAUC:↓ACC vs NOEX Pulse wave velocity: ↓ACC vs CONT; ↔ ACC vs NOEX
Yap et al. [20]	Randomized crossover	Healthy adults, *N* = 25 (M12/F13) Age: 23 ± 3 BMI: 21.0 ± 1.7	Accumulated: 3 × 10-min walking, interval: 20-min (Day 1) Continuous: 30-min walking after 40-min sitting (Day 1) Intensity @70% V̇O_2_max Rest: sit for 70-min (Day 1) Timing: all walking finished @Day1 18:10 h	Fasting insulin:↓Continuous vs Rest; ↔ Accumulated vs Rest; ↔ Continuous vs Accumulated; QUICKI:↓Continuous vs Rest; ↔ Accumulated vs Rest; ISI-Matsuda:↑Continuous vs Rest

Values are means ± SD or range (minimum–maximum); @, at; BMI, body mass index; F, females; HDL, high-density lipoprotein; HIIE, high-intensity interval exercise; HRmax, maximum heart rate; HRR, heart rate reserve; iAUC, incremental area under the curve; M, males; MET, metabolic equivalent of task; MOD, moderate-intensity; OGTT, oral glucose tolerance test; PA, physical activity; PPG, postprandial glucose; PPTG, postprandial triglyceride; QUICKI, quantitative insulin sensitivity check index; SIT, sitting; T2D, type 2 diabetes; TG, triglyceride; VIG, ﻿vigorous-intensity; V̇O_2_max, maximal oxygen uptake; V̇O_2_R, oxygen uptake reserve; ↑, increased; ↓, reduced; ↔ , no statistically significant difference between measures; < , less than; vs, compared with the following condition

**Table 2:** Long-term intervention study characteristics

Studies	Study Design & Duration	Participants (*N*, gender, age [year], BMI [kg/m^2^])	Intervention	Results
Asikainen et al. [38]	15 weeks randomized controlled trial	Postmenopausal women, *N* = 134 BMI ˂32; W1 (*n* = 46), Age: 58 ± 4.4; W2 (*n* = 43), Age: 58 ± 4.2; C1 (*n* = 45), Age: 57 ± 4.2;	W1: 1 × daily walking; Duration: 46.6 ± 5.4 min W2: 2 × daily walking with ≥ 5-h interval; Duration: 2 × 24.0 ± 3.2 min Walking @65% V̇O_2_max; TEE @300 kcal/d; 5 d/w C1: no daily walking Timing: anytime of the day	DBP: ↓ W1 & W2 vs C1 Mean blood glucose: ↓ W1 & W2 vs C1 2-h glucose: ↓ W1 & W2 vs C1 SBP, serum lipoproteins and insulin levels: ↔ in all groups
Eriksen et al. [31]	5 weeks randomized controlled trial	T2D, males, *N* = 28 Group 1 × 30: [*n* = 9; Age: 59 ± 8; BMI: 30(27–37)]; Group 3 × 10: [*n* = 9; Age: 60 ± 4; BMI: 35(29–41)]; Time-control: [*n* = 10; Age: 61 ± 7; BMI: 31(28–37)]	3 × 10-min: 3 × 10-min cycling/d 1 × 30-min: 1 × 30-min cycling/d Cycling @60–65% V̇O_2_max, 6-d/w Control group: Free-living conditions Physical activity and meal timing: unspecified	Cardiorespiratory fitness: ↑ Group 3 × 10 and 1 × 30 Fasting plasma glucose, 120-min glucose OGTT and PPG AUC at 120 and 180-min: ↓ Group 3 × 10, ↔ Group 1 × 30 ISI composite, ISR and Btotal: ↔ Group 3 × 10 and 1 × 30
Pahra et al. [15]	60-d randomized crossover trial	T2D, *N* = 64; Group A: (*n* = 32; Age:49.0 ± 9.7); Group B: (*n* = 32; Age: 50.7 ± 3.2)	Post-meal EX: 3 × 15-min walking, @15-min after each meal One-time daily EX: 1 × 45-min walking, before breakfast Walking @4.8 km/h	Five point blood glucose profile & HbA1c: ↓ Group A, B in Post-meal EX vs One-time daily EX
Reynolds et al. [14]	2 weeks randomized crossover trial	T2D, *N* = 41 (M26/F15); Age: 60 ± 9.9; BMI: ~ 32	Post-meal walking: 3 × 10-min walking, after each meal Single daily 30-min walking: 1 × 30-min walking, anytime of the day	PPG 3-h iAUC: ↓ Post-meal walking vs single daily 30-min walking

Values are mean ± SD or range (minimum–maximum); @, at; AUC, area under the curve; BMI, body mass index; DBP, diastolic blood pressure; EX, exercise; F, females; M, males; OGTT, oral glucose tolerance test; SBP, systolic blood pressure; T2D, type 2 diabetes; TEE, total energy expenditure; V̇O_2_max, maximal oxygen uptake; ↑, increased; ↓, reduced; ↔ , no statistically significant difference between measures; vs, compared with the following condition

### Risk of Bias Assessment

Two authors (XZ, CZ) independently performed the risk of bias assessment using the revised Cochrane risk-of-bias tool for randomized trials (RoB 2) [25]. Bias was assessed via RoB 2 in five distinct domains: randomization, deviations from intended interventions, missing outcome data, measurement, and selection of reported results [25]. Disagreements were resolved by discussion and clarified with a third reviewer (RH) if necessary.

### Data Synthesis and Analysis

Means and standard deviations (SD) were extracted from each study. For studies that reported standard errors or 95% CI, the SD was calculated as described in section 7.7.3.2 of the Cochrane Handbook [26]. The inverse variance random-effects method was used for all meta-analyses to combine data. All analyses were conducted using Review Manager Software (RevMan 5.4, Cochrane Collaboration, Copenhagen, Denmark). If a trial was used more than once for comparison with different trials in the meta-analysis, the sample size for that trial was divided by the number of times it was used [26]. For PPG or postprandial insulin, the total AUC was extracted; if both were provided, the incremental AUC was chosen. If data were missing, or only presented as a figure, the authors were contacted and asked to provide the relevant information. If this was unsuccessful, relevant data provided only in figures were extracted using WebPlotDigitizer 4.1 software (https://automeris.io/WebPlotDigitizer). To account for different measurement or time scales, continuous outcomes were analyzed using standardized mean differences (SMD) [26]. To improve the robustness of our findings, we conducted a series of sensitivity analyses to test the individual influence of each study, including those with a high risk of bias, on the overall results. With most studies being classified as with some concerns, sensitivity analyses were not performed on these studies. If at least ten trials were included in a meta-analysis, we investigated publication bias using funnel plots to explore the possibility of small study effects (i.e., a tendency for smaller studies to report larger beneficial effects). As accumulated exercise in the included studies could be summarized into two patterns, PA breaks and 2–3 short bouts timed around daily main meals, we performed subgroup analysis based on the number of exercise bouts [18]. One study showed that PA breaks may be better at attenuating PPG levels in young active individuals than three bouts of energy-matched exercise before or after each meal [27]. Previous reviews have examined the two patterns of accumulated exercise separately [18, 23] to ensure that interventions were sufficiently homogeneous for comparison, or included studies with PA breaks only [17, 19]. Subgroup analysis was also performed for exercise intensity, as this may affect PPG and insulin responses [23, 28]. Populations with or without diabetes were selected as another subgroup characteristic, given that metabolic responses to exercise might be different in individuals with a different glucose status [17, 28].

### Assessment of Heterogeneity

Meta-analyses were performed with Review Manager Software (RevMan 5.4, Cochrane Collaboration, Copenhagen, Denmark) when data were available from two or more trials. For outcomes with insufficient available data to pool, we presented the results individually. All heterogeneity was examined through the Chi-square test and we also used the *I*^2^ statistic, indicating the percentage of the variability that is due to heterogeneity rather than chance (< 25%, low heterogeneity; 26–50%, moderate heterogeneity; > 50%, high heterogeneity) [29].

## Results

### Description of Studies

A total of 5183 individual studies were identified through the initial search process after the removal of duplicates. Thirty-one studies underwent full-text review, and 9 out of the 31 studies were excluded. Studies were excluded according to the following exclusion criteria: (1) did not meet intervention or comparator criteria (*n* = 3, e.g., combined continuous and accumulated exercise, accumulated exercise in a single session, such as HIIT); (2) only the abstract was available (*n* = 3); (3) did not meet outcome criteria (*n* = 2, e.g., did not provide any of the outcomes of interest, including glucose-, insulin-, or TG-related measures); and (4) duplicates (*n* = 1). Three studies were identified from citation searching, and two studies were included from the updated search. Therefore, a total of 27 studies were included. The results of the systematic search are presented in Fig. 1. Below we provide a summary of the key characteristics (participants, study design, intervention, and outcome details) of these eligible studies (see Tables 1, 2 for an overview of short-term and long-term intervention study characteristics, respectively).

#### Study Designs

Of the 27 studies that met our inclusion criteria, four studies [14, 15, 30, 31] with 267 participants were long-term (≥ 2 weeks) intervention studies, with a duration of 2–15 weeks. All 23 short-term studies and two long-term intervention studies were randomized crossover design, whereas two long-term intervention studies were parallel randomized controlled trials [30, 31]. There were 13 out of the 23 short-term studies with a total of 118 participants utilized 1-day designs and examined the effect of exercise within the same day, with durations ranging from 4-h to 24-h, while ten studies with 250 participants utilized multi-day designs and examined the effect of exercise on the second-morning responses, including one study [13] that examined both the same-day and second-morning effect.

#### Participants

A total of 27 studies with 635 participants were included in the meta-analysis. Three of the long-term studies [14, 15, 31], a total of 133 participants, and three of the short-term studies [32–34], a total of 48 participants, recruited individuals with type 2 diabetes. In addition, one short-term study [16] included adults with insulin resistance (*n* = 9); all other studies were conducted in non-diabetic populations. Most of the participants were physically inactive or sedentary. Five [34–38] and seven [31, 33, 39–43] studies included only females and males, respectively. The average age of participants ranged from 22.6 [44] to 71.0 years [36]; the body mass index (BMI) ranged from 21.0 [20] to 34.0 kg/m^2^ [45]. Sample sizes ranged from 8 [41] to 134 [38].

#### Interventions

Accumulated exercise in the included studies could be characterized into two types: (1) 2–3 bouts of exercise (e.g., 10–15 min per bout) timed around meals; and (2) activity spread across frequent, brief bouts (≥ 5 bouts, e.g., 1–6 min per bout) throughout the day (known as PA breaks). Thirteen studies adopted PA breaks, while 14 studies (including four long-term intervention studies) adopted 2–3 bouts of accumulated exercise. For PA breaks, most studies (*n* = 12) used frequent brief bouts with 1–5 min per bout every 15–60 min in a 6.5–12 h period, except for one study [35] that included 5 bouts of 6 min cycling within 240 min. Moreover, except for four studies [35, 43, 44, 46] involving high-intensity exercise, most PA breaks (*n* = 9) involved low-moderate intensity exercise. For 2–3 bouts of exercise, most studies adopted 3 bouts of 10–15 min exercise (*n* = 11); three studies adopted 2*20 min [33], 3*30 min [39], and 2*24 min [30] accumulated exercise. Except for one study [16] involving high intensity exercise, all 2–3 bouts of exercise involved low-moderate intensity exercises. Most short bouts of exercise were before or after each main meal, with an interval of 4–5-h (*n* = 9), except for two studies with an interval of 20 min [20, 47]; one study conducted 2 bouts of 20 min walking before and after 40 min of lunch [33], while two long-term studies did not specify exercise timing or intervals [30, 31]. For energy-matched continuous exercise, except for a study involving one session of high-intensity interval exercise [44], all other studies used one bout of 30–90 min low-moderate intensity exercise. Two studies [48, 49] were excluded due to the unmatched energy expenditure.

#### Outcomes

Nineteen short-term studies, a total of 295 participants, reported PPG AUC indices. Of these studies, 12 comprising 210 participants, reported the same-day effect, while the remaining seven reported the second-morning effect. Four studies recruited individuals with type 2 diabetes, while the remaining 15 studies recruited non-diabetic populations. Moreover, 12 studies, comprising 214 participants, compared the effects of PA breaks with those of continuous exercise on PPG AUC, while the remaining seven studies, a total of 81 participants, compared 2–3 bouts of accumulated exercise with continuous exercise. Four studies included high-intensity exercise; five and three studies recruited only men and women, respectively. Six short-term studies, comprising 78 participants, reported 24-h glucose indices (24-h glucose AUC or 24-h mean glucose) measured using continuous glucose monitoring. Five short-term studies reported second-morning fasting glucose [13, 20, 41–43], while all four long-term intervention studies reported PPG AUC and fasting glucose.

Postprandial insulin AUC was reported in 12 studies, comprising 202 participants, of which, six studies examined the same-day effect. Fasting insulin levels were reported in seven studies with second-morning effects and two long-term intervention studies [31, 38].

Moreover, postprandial TG was reported in 13 studies, a total of 227 participants, of which, eight examined the same-day effect. Two and four long- and short-term intervention studies, respectively, reported fasting TG.

### Intervention Effects

#### Glucose Measures

**Fig. 2:**
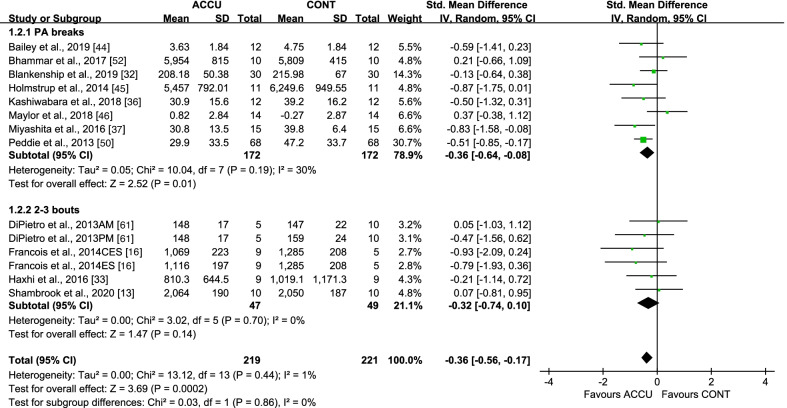
Effect of accumulated versus continuous exercise on same-day postprandial glucose, stratified by exercise bouts. ACCU, accumulated exercise; CONT, continuous exercise; PA, physical activity. PA breaks represents frequent brief bouts (e.g., 1–6 min) throughout the day at 20–60-min intervals (≥ 5 bouts); 2–3 bouts represents 2–3 short bouts of accumulated exercise. AM, 45 min of sustained walking performed at 10:30 a.m; PM, 45 min of sustained walking performed at 4:30 p.m; CES, composite exercise snacking; ES, exercise snacking

**Fig. 3:**
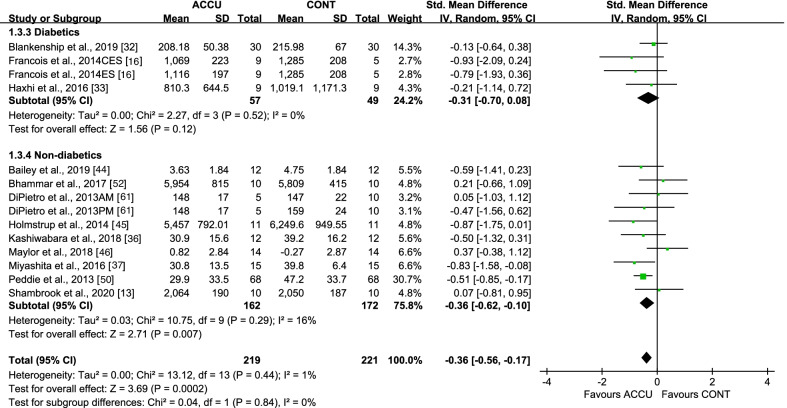
Effect of accumulated versus continuous exercise on same-day postprandial glucose, stratified by population. ACCU, accumulated exercise; CONT, continuous exercise. AM, 45 min of sustained walking performed at 10:30 a.m; PM, 45 min of sustained walking performed at 4:30 p.m; CES, composite exercise snacking; ES, exercise snacking

**Fig. 4:**
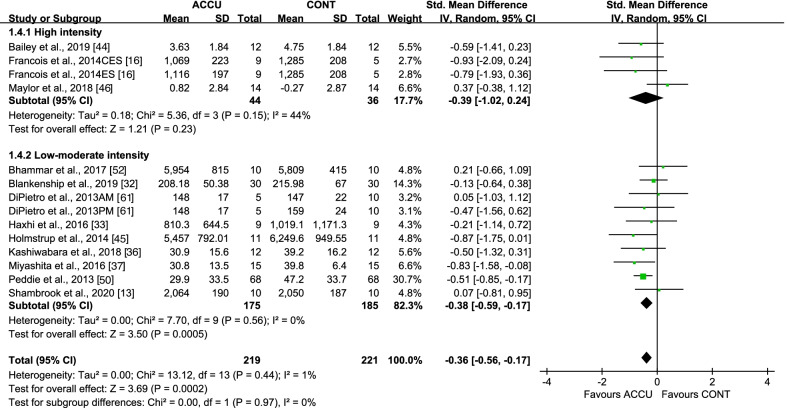
Effect of accumulated versus continuous exercise on same-day postprandial glucose, stratified by exercise intensity. ACCU, accumulated exercise; CONT, continuous exercise. AM, 45 min of sustained walking performed at 10:30 a.m; PM, 45 min of sustained walking performed at 4:30 p.m; CES, composite exercise snacking; ES, exercise snacking

When considering short-term effects, some studies were designed to examine the same-day effects of the exercise intervention, while others examined these effects the following day via second-morning responses. For same-day effects, accumulated, compared to continuous, exercise showed a significant lowering effect on PPG AUC, with an SMD of − 0.36 (95% CI: [− 0.56, − 0.17], *P* = 0.0002, *I*^2^ = 1%) (Fig. 2). Subgroup analysis indicated that only PA breaks as accumulated exercise (SMD − 0.36 [95% CI: (− 0.64, − 0.08)], *P* = 0.01, *I*^2^ = 30%) reduced PPG AUC, compared to continuous exercise, while 2–3 bouts of accumulated exercise did not (SMD − 0.32 [95% CI: (− 0.74, 0.10)], *P* = 0.14, *I*^2^ = 0%) (Fig. 2). Moreover, accumulated, compared to continuous, exercise had a greater effect on PPG AUC in studies with nondiabetic populations (SMD − 0.36 [95% CI: (− 0.62, − 0.10)], *P* = 0.007, *I*^2^ = 16%) but not in studies with diabetic populations (Fig. 3), and in studies with low- to moderate-intensity exercise (SMD − 0.38 [95% CI: (− 0.59, − 0.17)], *P* = 0.0005, *I*^2^ = 0%), but not in studies with high-intensity exercise (Fig. 4). All studies measuring 24-h glucose (24-h mean glucose or 24-h glucose AUC) focused on same-day effects. Furthermore, 24-h glucose measures showed no significant differences between accumulated and continuous exercise (SMD − 0.21 [95% CI: (− 0.52, − 0.09)], *P* = 0.17, *I*^2^ = 0%) (Additional file 1: Fig. S1).

**Fig. 5:**
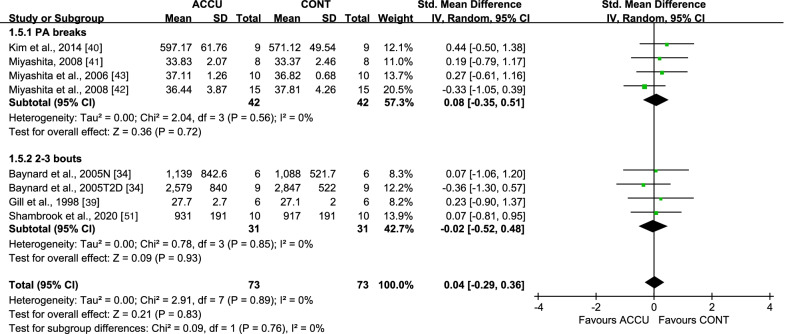
Effect of accumulated versus continuous exercise on second-morning postprandial glucose, stratified by exercise bouts. ACCU, accumulated exercise; CONT, continuous exercise. PA breaks represents frequent brief bouts (e.g., 1–6 min) throughout the day at 20–60-min intervals (≥ 5 bouts); 2–3 bouts represents 2–3 short bouts of accumulated exercise. N, non-obese; T2D, type 2 diabetes

For second-morning effect on PPG AUC, no significant differences were observed between accumulated and continuous exercise (SMD 0.04 [95% CI: (− 0.29, 0.36)], *P* = 0.83, *I*^2^ = 0%) (Fig. 5). Moreover, no differences in exercise bout-based subgroup analysis were observed (Fig. 5). As less than two studies focused on high-intensity exercise or diabetic populations, no exercise intensity- and population-based subgroup analyses were performed. No differences were observed between accumulated and continuous exercise effects on second-morning fasting glucose (SMD − 0.07 [95% CI: (− 0.19, 0.06)], *P* = 0.29, *I*^2^ = 14%) (Additional file 1: Fig. S2).

For long-term intervention studies, no differences in PPG AUC (SMD − 0.55 [95% CI: (− 1.47, 0.37)], *P* = 0.24, *I*^2^ = 93%) (Additional file 1: Fig. S3) and fasting glucose (SMD − 0.46 [95% CI: (− 1.30, 0.37)], *P* = 0.28, *I*^2^ = 92%) (Additional file 1: Fig. S4) were observed between exercise conditions.

#### Insulin Measures

**Fig. 6:**
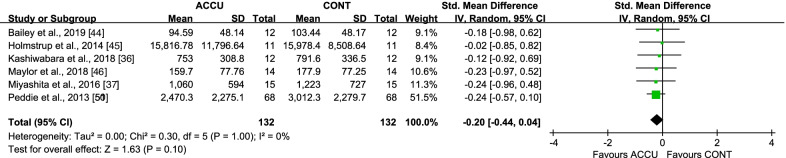
Effect of accumulated versus continuous exercise on same-day postprandial insulin. ACCU, accumulated exercise; CONT, continuous exercise

For short-term effects, no differences were observed between accumulated and continuous exercise effects on either same-day postprandial insulin AUC (SMD − 0.20 [95% CI: (− 0.44, 0.04)], *P* = 0.10, *I*^2^ = 0%) (Fig. 6) or second-morning postprandial (SMD − 0.29 [95% CI: (− 0.74, 0.15)], *P* = 0.20, *I*^2^ = 28%) (Additional file 1: Fig. S5) and fasting insulin (SMD − 0.06 [95% CI: (− 0.37, 0.24)], *P* = 0.69, *I*^2^ = 0%) (Additional file 1: Fig. S6).

Only two long-term intervention studies measured insulin levels, with no differences observed on either postprandial insulin AUC (SMD 0.18 [95% CI: (− 0.21, 0.56)], *P* = 0.36, *I*^2^ = 0%) or fasting insulin (SMD 0.07 [95% CI: (− 0.55, 0.69)], *P* = 0.82, *I*^2^ = 43%) outcomes.

#### TG Measures

**Fig. 7:**
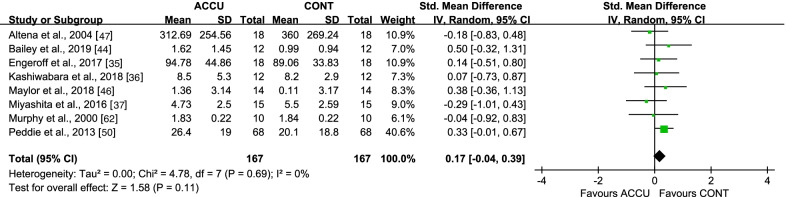
Effect of accumulated versus continuous exercise on same-day postprandial triglycerides. ACCU, accumulated exercise; CONT, continuous exercise

For short-term effects, no differences were observed for same-day postprandial TG AUC (SMD 0.17 [95% CI: (− 0.34, 0.39)], *P* = 0.11, *I*^2^ = 0%) (Fig. 7), second-morning postprandial TG AUC (SMD 0.11 [95% CI: (− 0.24, 0.47)], *P* = 0.53, *I*^2^ = 0%) (Additional file 1: Fig. S7), and second-morning fasting TG (SMD − 0.08 [95% CI: (− 0.51, 0.35)], *P* = 0.73, *I*^2^ = 0%) (Additional file 1: Fig. S8). The pooled effects from two long-term intervention studies showed no difference in fasting TG (SMD 0.22 [95% CI: (− 0.16, 0.61)], *P* = 0.25, *I*^2^ = 0%).

### Risk of Bias

**Fig. 8:**
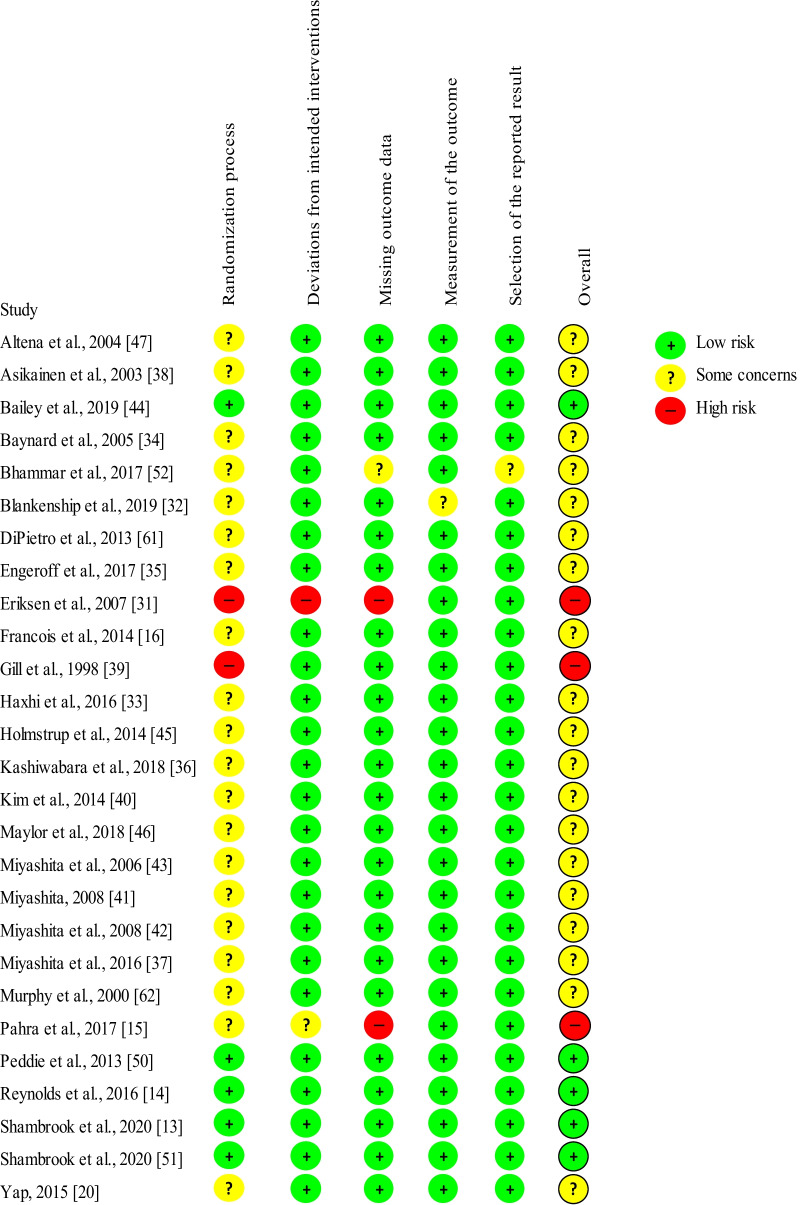
Risk of bias summary

Overall, most of the studies were with some concerns, excluding three studies with a high risk of bias [15, 31, 39] and five with a low risk of bias [13, 14, 44, 50, 51] (Fig. 8). Except for two studies that did not specify whether they were randomized trials [35, 39], all other studies were randomized trials. Of these, only seven reported randomization details [13, 14, 38, 44, 46, 50, 51]. Moreover, only five studies clearly reported that participants were blinded until arriving at the laboratory to complete the trials [13, 14, 44, 50, 51]. Although the study design does not enable participants or research staff to be blinded from the intervention and the measurement of the outcomes, the measured outcomes are difficult for either the participant or researcher to influence. Most studies did not report whether there were any missing data and how the missing data were handled. Two studies [15, 31] were considered as having a high risk of bias and one study [52] as with some concerns due to missing outcome data. One study [31] was defined as a “per-protocol” effect protocol, as one participant was excluded for not adhering to the exercise intensity protocol. Only one study was with some concerns for baseline imbalances due to differences in baseline BMI between groups [31]. Most crossover studies had at least a 3-day wash-out period between trials to avoid the carry-over effect; one study [32] had only a 1-day wash-out period.

### Sensitivity Analyses and Publication Bias

Sensitivity analyses, in which studies with a high risk of bias were removed, did not substantially change the results. A series of sensitivity analyses were performed by removing each of the studies. This showed that when only one study [50] was removed, the subgroup analyses favoring the effects of accumulated exercise with PA breaks (Fig. 2), or in non-diabetic populations (Fig. 3), on same-day PPG AUC were no longer significant. Only the same-day effect on PPG AUC had at least 10 studies, a necessary requirement for conducting a publication bias assessment; funnel plots showed no indication of publication bias (Additional file 1: Fig. S9).

## Discussion

Accumulated exercise produced a greater effect on same-day PPG control than one bout of energy-matched continuous exercise. This greater effect was especially observed in subgroups of accumulated exercise with PA breaks (≥ 5 bouts), low-moderate intensity exercise, and in non-diabetic populations. However, no differences were found in second-morning PPG control and fasting glucose, insulin, and TG. Moreover, no differences were observed in postprandial insulin and TG between continuous and accumulated exercise, both for same-day or second-morning effects.

Our findings are consistent with two previous reviews, which showed that PA breaks had a greater effect on glycemia when compared with energy-matched continuous exercise, with a SMD of − 0.26 (95% CI: [− 0.50, − 0.02], *P* = 0.03) [17] and − 0.386 [95% CI: (− 0.718, − 0.054)], *P* = 0.023) [19]. The major differences between the present and previous meta-analyses include that we examined same-day and second-morning effects separately, and included two patterns of accumulated exercise, i.e., PA breaks and 2–3 short bouts of exercise. Our findings suggest that although PA breaks can reduce the PPG response on the same day, compared to a single-session of exercise, it has no benefits on the second-morning response, which reflects the residual effects 12–24 h afterward. This finding is consistent with a previous systematic review and meta-analysis [23] showing that compared to prolonged sitting, activity breaks lowered PPG on the same, but not the following, day after activity.

Although there was one narrative review [18], no meta-analysis has compared 2–3 short bouts, with a single bout, of exercise on PPG. Chang et al. [18] suggested that three short bouts of accumulated, compared with a single bout of continuous, activity lead to similar or superior effects on glycemic control in individuals with prediabetes and type 2 diabetes. Our meta-analysis showed no differences in either same-day or second-morning PPG responses between 2–3 bouts of exercise and a single bout of energy-matched exercise. Our exercise intensity-focused subgroup analysis showed that accumulated exercise produced greater effects at low-moderate intensity only, but not high-intensity, which may induce a glucose counterregulation response [19]. Another underlying mechanism may be that greater energy expenditure occurs from multiple short bouts, compared to one bout, of exercise due to an acute increase in exercise-induced metabolic rate related to excess post-exercise oxygen consumption [53, 54]. Although subgroup analysis indicated that only non-diabetic populations gain benefits to PPG from accumulated exercise, compared to continuous exercise, it is possible that the small number of studies with diabetic populations eligible for subgroup analysis limited the statistical power to detect small differences.

Although interrupting prolonged sitting with PA breaks has been clearly shown to produce marked and meaningful improvements in PPG and insulin metabolism [17, 23, 55], the effectiveness of accumulated exercise compared to a single session of energy-matched continuous exercise has not been well established [13, 18]. Our findings suggest that accumulated exercise is an effective and feasible alternative for gaining health benefits; such activities can be even more meaningful for those who are sedentary and spend limited time doing exercise in one session [56]. Given that elevated PPG, postprandial insulin, and TG are independent and important risk factors for chronic disease morbidity and all-cause mortality [1, 2, 57, 58], the glucose-lowering effect of accumulated exercise may be clinically relevant if experienced on a regular basis. The reason for no difference of TG is potentially that the total energy expenditure of exercise is the primary determinant of the exercise-induced reductions in postprandial TG [59] irrespective of the pattern of exercise. Regarding insulin, the small studies included in this analysis had findings that were difficult to generalize in terms of the effects (if any) of their differing activity patterns, particularly for moderate-intensity exercise with relatively normal duration in healthy young adults.

Overall, based on the risk of bias assessment, as most of the studies provided insufficient randomization and blinding details, they were classified as with some concerns. Future studies should report information regarding randomization, blinding, and missing data more clearly. Sensitivity analyses removing the studies with a high risk of bias did not affect any of the results (data not shown). Only four long-term intervention studies were included in this review, with two [15, 31] of these identified as having a high risk of bias; more long-term intervention studies with a low risk of bias are warranted in the future. Sensitivity analysis by removing Peddie et al. [50] affected the results of subgroups analyses, because this study was heavily weighted due to the large sample size. Peddie et al. [50] conducted a well-controlled and -designed randomized crossover study, compared regular PA breaks to one session of 30-min walking, with a relatively large sample size (*n* = 70), and with a duration of 9 h. It is possible that the sample size, duration of experiment, the frequency and intensity of the PA breaks, and the intensity and duration of continuous exercise may affect the results regarding the difference between accumulated and continuous exercise.

There are several strengths to this systematic review and meta-analysis. First, we used a comprehensive search strategy to include different types of accumulated exercise, namely PA breaks and 2–3 bouts, in both short- and long-term interventions, and diabetic and non-diabetic populations. This approach allowed us to analyze same-day and second-morning effects separately to reduce study design heterogeneity, as well as enabled us to perform exercise bout-, intensity-, and population-based subgroup analyses to explore potential factors that may affect the results. Furthermore, as all studies included energy-matched exercises, direct comparisons between exercise conditions were possible.

This review has several limitations. The timing of exercise in relation to a meal may be an important factor affecting glucose regulation results [12, 13, 18]; however, exercise timing varied between accumulated and continuous exercises in most of the included studies, demonstrating the potential confounding effect of meals. Moreover, the measurement scales were heterogeneous; incremental or total AUC was calculated using different time scales, and either continuous glucose monitoring (CGM) or venous blood glucose was considered. However, as these results are strongly correlated [60], combining the methods was acceptable, and allowed us to maximize the available data [23]. In addition, some selection bias may be present as only published peer-reviewed studies in the English language were included. Furthermore, as limited long-term intervention studies with high heterogeneity were identified, caution should be exercised in interpreting these data.

## Conclusion

Compared with one bout of continuous exercise, accumulated exercise produced a greater acute effect on same-day PPG control. This greater effect was especially observed in subgroups of accumulated exercise with PA breaks, low-moderate intensity exercise, and in non-diabetic populations. No differences were observed in second-morning glucose control. Moreover, no differences in postprandial and fasting insulin and TG were observed between continuous and accumulated exercise interventions. Due to the limited number of long-term intervention studies with a varied risk of bias, it was difficult to conclude any long-term effects. Future studies are required to investigate the long-term effects of accumulated versus continuous exercise on postprandial metabolism.

## Supplementary Information

**Additional file 1**. Table S1 and Fig. S1–S9.

## Data Availability

All data generated or analyzed during this study are included in this published article and its supplementary information files.

## Abbreviations

ACCU: Accumulated exercise
CONT: Continuous exercise
HIIT: High-intensity intermittent exercise
PA: Physical activity
PPG: Postprandial glucose
SD: Standard deviations
SMD: Standardized mean differences
TG: Triglyceride
