# Supplementary material for: The Effects of Accumulated Versus Continuous Exercise on Postprandial Glycemia, Insulin, and Triglycerides in Adults with or Without Diabetes: A Systematic Review and Meta-Analysis
Source: Sports Med Open. 2022 Jan 24;8:14. doi: 10.1186/s40798-021-00401-y (PMC8786998; doi:10.1186/s40798-021-00401-y)
Supplement: Supplementary file 1 — Additional file 1. Table S1 and Fig. S1–S9. [file 40798_2021_401_MOESM1_ESM.docx]

**Additional file 1**

**Title: The effects of accumulated versus continuous exercise on postprandial glycemia, insulin, and triglycerides in adults: a systematic review and meta-analysis**

Xiaoyuan Zhang, Chen Zheng, Robin S.T. Ho, Masashi Miyashita, Stephen H.S. Wong*

* Corresponding Author: Stephen H.S. Wong, Ph.D.

Department of Sports Science and Physical Education, The Chinese University of Hong Kong, Hong Kong, China; Email: [hsswong@cuhk.edu.hk](mailto:hsswong@cuhk.edu.hk)

**Table S1 Search strategy (Medline^1^)**

| 1. Search strategy for “**randomized controlled trial”**   ((clinical[Title/Abstract] AND trial[Title/Abstract]) OR clinical trials as topic[MeSH Terms] OR clinical trial[Publication Type] OR random*[Title/Abstract] OR random allocation[MeSH Terms] OR therapeutic use[MeSH Subheading]) |
| --- |
| 1. Search strategy for “**exercise”**   ((((exercise[MeSH Terms]) OR (exercis*[Title/Abstract]) OR  (physical activit*[Title/Abstract]) OR (physical activity[MeSH Terms]) OR  (walk*[Title/Abstract]) OR motor activity  (sports[MeSH Terms]) OR (sport*[Title/Abstract]) OR (fitness[Title/Abstract]) OR  (resistance training[MeSH Terms]) OR (weight training[Title/Abstract]) OR (weightlifting[Title/Abstract]) OR  (running[Title/Abstract]) OR (*cycling[Title/Abstract]) OR (swimming[Title/Abstract])) OR jog* |
| 1. Search strategy for **“accumulated” or “continuous”**   (continuous[Title/Abstract]) OR (Continu*) OR  ((single bout*[Title/Abstract]) OR (long bout*[Title/Abstract]) OR (one bout*) OR  (multiple bout*[Title/Abstract]) OR (short* AND bout*[Title/Abstract]) OR (repeated bout*[Title/Abstract]) OR (accumulat*[Title/Abstract]) OR  (physical activity pattern*[Title/Abstract]) OR (exercise pattern*[Title/Abstract])) OR  “breaks” OR “breaking” OR “break*" OR  "interrupting" OR (intermittent[Title/Abstract]) OR  “intervals” OR "alternating" OR “Exercise snack*” OR “periodic exercise” |
| 1. Search strategy for “**glucose”** **“insulin” or “lipid”**   "glucose" OR "glycaemia" OR "glycemia" OR “*Glyc*mi*” OR  “Glucose intoleran*” OR  “*Insulin*” OR “Insulin sensitivity” OR “Insulin resistance” OR  "lipid*" OR "lipaemia" OR "lipemia" OR "*lip*mi*" OR  "triglycerides" OR "triglyceride*" OR “triacylglycerol” OR “triacylglyceride” |
| 1. Search strategy for “**postprandial”**   “postprandial” OR “post*prandial*” OR “post prandial” OR “postprandial period” OR “post*meal” OR “post meal” OR  post challenge[Title/Abstract]) OR postchallenge[Title/Abstract]) OR post-challenge[Title/Abstract]) OR post*challenge OR “post-load” OR peak[Title/Abstract]) OR Glucose Tolerance Test* OR OGTT OR “tolerance test*” OR “meal test*”  AUC[Title/Abstract] OR iAUC[Title/Abstract] OR area under the curve[Title/Abstract]) OR area under curve[Title/Abstract])  meal* OR breakfast OR lunch OR dinner OR drink* OR [beverage*](http://ovidsp.dc1.ovid.com/ovid-b/ovidweb.cgi?&Controlled+Vocabulary=Mapping%7c8&Return=mapping&S=FIFHFPNEHNACELLCKPAKLEDKHOJCAA00) OR  OR continuous glucose monitoring OR interstitial glucose |

^1^ The search strategy was initially designed for Medline and was adapted for searching the other databases. Keywords were combined within-terms using the ‘OR’ operator, and the final search phrase was conducted by combining the five search terms using the ‘AND’ operator.


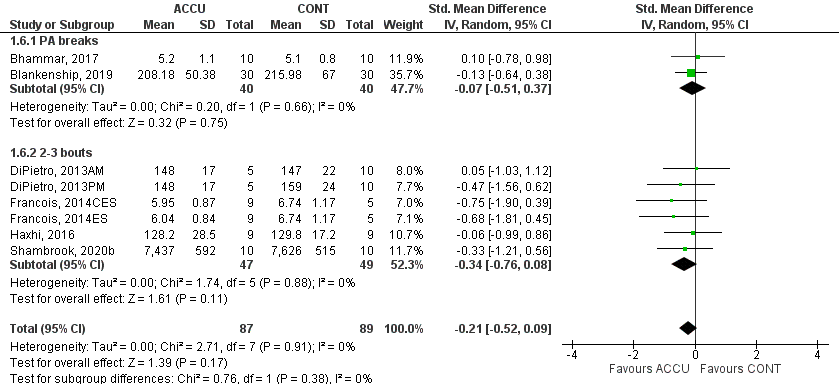


**Fig.S1.** Forest plot of comparison between accumulated and continuous exercise on 24-h glucose measures. ACCU, accumulated exercise; CONT, continuous exercise; PA breaks represents frequent brief bouts (e.g., 1–6 min) throughout the day at 20–60-min intervals (≥5 bouts); 2-3 bouts represents 2-3 short bouts of accumulated exercise.


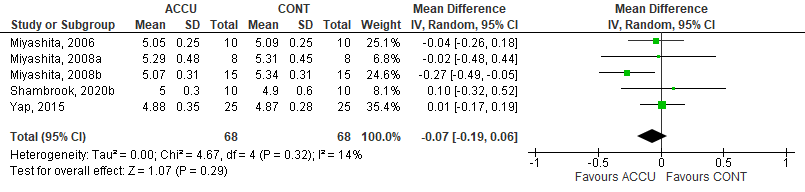


**Fig.S2.** Forest plot of comparison between accumulated and continuous exercise on second-morning fasting glucose. ACCU, accumulated exercise; CONT, continuous exercise.


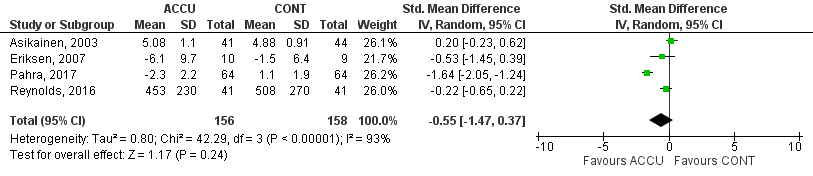


**Fig.S3.** Forest plot of comparison between long-term accumulated and continuous exercise intervention on postprandial glucose. ACCU, accumulated exercise; CONT, continuous exercise.


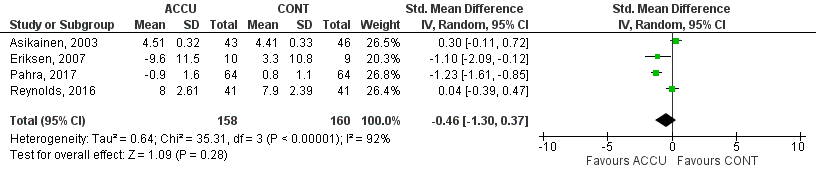


**Fig.S4.** Forest plot of comparison between long-term accumulated and continuous exercise intervention on fasting glucose. ACCU, accumulated exercise; CONT, continuous exercise.


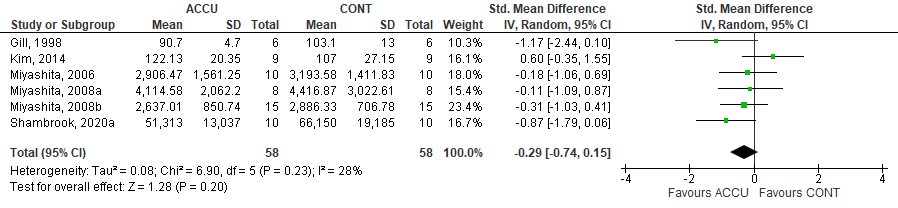


**Fig.S5.** Forest plot of comparison between accumulated and continuous exercise on second-morning postprandial insulin. ACCU, accumulated exercise; CONT, continuous exercise.


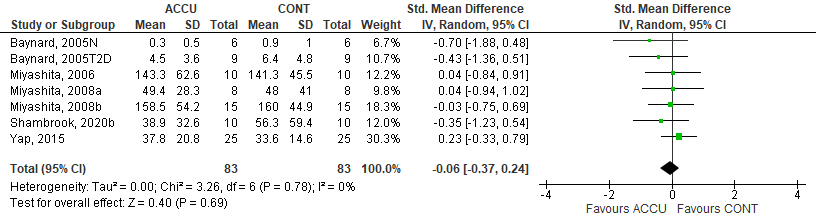


**Fig.S6.** Forest plot of comparison between accumulated and continuous exercise on second-morning fasting insulin. ACCU, accumulated exercise; CONT, continuous exercise.


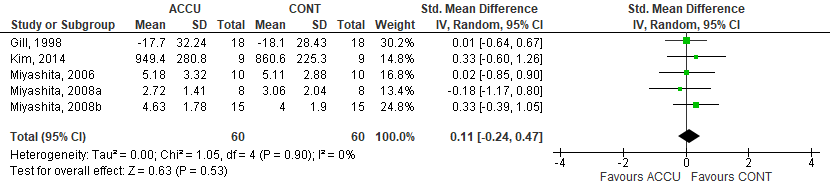


**Fig.S7.** Forest plot of comparison between accumulated and continuous exercise on second-morning postprandial triglycerides. ACCU, accumulated exercise; CONT, continuous exercise.


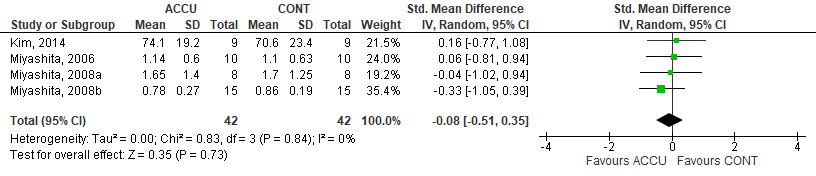


**Fig.S8.** Forest plot of comparison between accumulated and continuous exercise on second-morning fasting triglycerides. ACCU, accumulated exercise; CONT, continuous exercise.


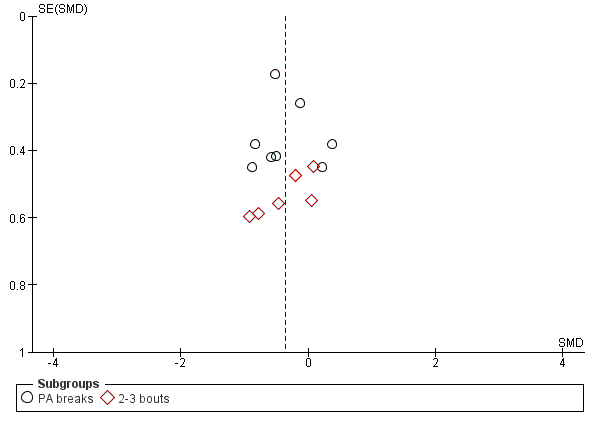


**Fig.S9.** Funnel plot of comparison between accumulated and continuous exercise on same-day postprandial glucose, stratified by exercise bouts. PA breaks represents frequent brief bouts (e.g., 1–6 min) throughout the day at 20–60-min intervals (≥5 bouts); 2-3 bouts represents 2-3 short bouts of accumulated exercise.
